# miR-125b develops chemoresistance in Ewing sarcoma/primitive neuroectodermal tumor

**DOI:** 10.1186/1475-2867-13-21

**Published:** 2013-03-04

**Authors:** Keiichiro Iida, Jun-ichi Fukushi, Yoshihiro Matsumoto, Yoshinao Oda, Yusuke Takahashi, Toshifumi Fujiwara, Yuko Fujiwara-Okada, Mihoko Hatano, Akira Nabashima, Satoshi Kamura, Yukihide Iwamoto

**Affiliations:** 1 Department of Orthopaedic Surgery, Graduate School of Medical Sciences, Kyushu University, Maidashi3-1-1, Fukuoka, 812-8582, Japan; 2 Department of Anatomical Pathology, Graduate School of Medical Sciences, Kyushu University, Maidashi3-1-1, Fukuoka, 812-8582, Japan

**Keywords:** Ewing sarcoma/primitive neuroectodermal tumor, miR-125b, p53, Bak, Chemoresistance

## Abstract

**Background:**

Diverse functions of microRNAs (miRNAs), including effects on tumorigenesis, proliferation, and differentiation, have been reported, and several miRNAs have also been demonstrated to play an important role in apoptosis. In this study, we investigated the possible role that miRNAs may play in the development of chemoresistance in Ewing sarcoma/primitive neuroectodermal tumor (EWS).

**Methods:**

We screened doxorubicin (Dox)-resistant EWS cells to identify any distinct miRNA sequences that may regulate the chemoresistance of EWS cells. The effects of miRNAs were evaluated using a chemosensitivity assay. The possible target genes of the miRNAs were predicted using a web-based prediction program.

**Results:**

We found miR-125b to be upregulated in two different Dox-resistant EWS cell lines. The upregulation of miR-125b was also confirmed in the EWS tumors having survived chemotherapy regimen which includes doxorubicin. When miR-125b was knocked down in EWS cells, both the Dox-resistant and parental cells showed an enhanced sensitivity to doxorubicin, which was associated with the upregulation of the pro-apoptotic molecules, p53 and Bak. Inversely, the overexpression of miR-125b in parental EWS cells resulted in enhanced drug resistance, not only to doxorubicin, but also to etoposide and vincristine.

**Conclusions:**

Our findings suggest that miR-125b may play a role in the development of chemoresistance in EWS by suppressing the expression of the apoptotic mediators, such as p53 and Bak.

## Background

The Ewing sarcoma/primitive neuroectodermal tumor (EWS) is a malignant small round cell tumor of the bone and soft tissues, and ranks second in frequency among primary bone tumors in children and adolescents. EWS is aggressive, and is associated with the most unfavorable prognosis of all primary musculoskeletal tumors [1]. With the development of multimodal therapeutic regimens that include chemotherapy, irradiation, and surgery, long-term survival has been achieved for approximately 70% of patients with localized disease [2]. However, smaller improvements have been observed for patients with recurrent disease, largely because their tumors are resistant to chemotherapy [3-6].

Several different mechanisms of chemoresistance in cancer have been elucidated. Enhanced drug-efflux pump activity, changes in the intracellular metabolic machinery, upregulation of DNA repair mechanisms, induction of growth signaling, and impairment of apoptosis can all lead to the acquisition of drug resistance [7]. In EWS, insulin-like growth factor [8,9], c-kit [10,11], CD99 [12], CD133 [13], and p53 [14] have been reported to modulate the anti-tumor effects of chemotherapy. We have previously reported that P-glycoprotein (P-gp) and MRP1 were overexpressed in doxorubicin (Dox)-resistant EWS cells, and the cells treated with P-gp and MRP inhibitors showed improved sensitivity to various drugs [15,16].

In addition to the above mechanisms, recent studies have focused on the involvement of microRNA (miRNA) during the acquisition of chemoresistance in cancer. The miRNAs are small endogenous non-coding RNAs that downregulate gene expression mainly by binding to the 3′UTR of the target gene region [17]. Their diverse functions, including effects on tumorigenesis, proliferation, differentiation, and apoptosis have been reported [18], and several miRNAs have been shown to have an important role in the development of chemoresistance [19]. Very recently, miR-34a was reported to regulate the chemosensitivity of EWS cells and to be a prognostic marker [20].

In the present study, we screened Dox-resistant EWS cells for distinct miRNA sequences that may regulate the chemosensitivity of EWS cells. Among 46 miRNAs, miR-125b was commonly upregulated in two different Dox-resistant cell lines. The upregulation of miR-125b was also confirmed in the EWS tumors having survived chemotherapy regimen which includes doxorubicin. We demonstrate that miR-125b promotes multidrug resistance by suppressing the expression of two apoptotic mediators, p53 and Bak. Further, our observations indicate that the miR-125b-p53/Bak pathway plays a role during the acquisition of Dox-resistance, and may potentially serve as a therapeutic target for EWS.

## Results

### miR-125b enhances the chemoresistance of EWS cells to doxorubicin

**Figure 1:**
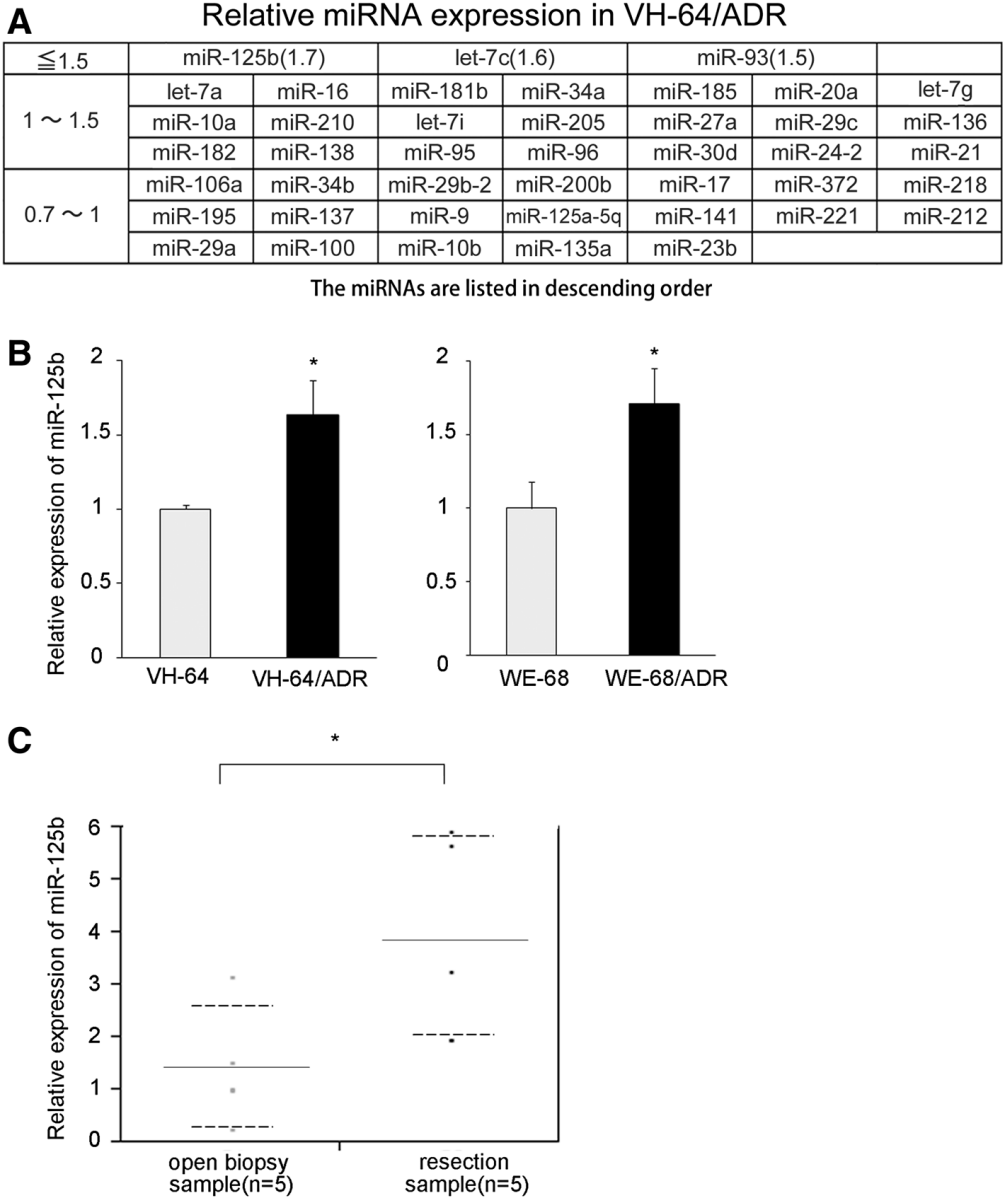
**The miRNA expression levels in Dox-resistant EWS cells. A**) The relative miRNA expression in Dox-resistant VH-64/ADR compared to parental VH-64 cells. The 46 miRNAs are listed in descending order. **B**) qRT-PCR was performed to confirm the differences in the expression of miR-125b in two different Dox-resistant cell lines. RNU6B was used as an internal control. The data represent the means of three separate experiments. The results are the means ± SD. *, P < 0.05. **C**) qRT-PCR was performed to confirm the differences in the expression of miR-125b in EWS tumors before and after treatment. A comparison was made between the EWS tumors before (open biopsy sample) and after (resection sample) VDC-IE or VAIA chemotherapy. RNU6B was used as an internal control. The gene expression levels were normalized to the average miR-125b expression level of the open biopsy samples. The results are given as the means (solid lines) ± SD (dashed lines). *, P < 0.05.

We examined the expression of miRNAs in Dox-resistant EWS cells (VH-64/ADR) using the Luminex multiplex assay system, and compared the results with those observed in the parental VH-64 cells. Among the 46 different miRNAs examined, miR-125b was the most highly upregulated miRNA in the VH-64/ADR cells compared to the parental cells (Figure 1A). The upregulation of miR-125b in the resistant cells was then confirmed by quantitative RT-PCR (the expression was 1.6-fold higher than that of the parental cells) (Figure 1B). The upregulation of miR-125b was also observed in another Dox-resistant EWS cell line, WE-68/ADR (also 1.6-fold higher than the parental WE-68 cells) (Figure 1B).

We were interested whether miR-125b was expressed in human EWS tumors. We examined five EWS tumor sample pairs, consisting of samples collected before and after chemotherapy using a VDC-IE (vincristine, doxorubicin, cyclophosphamide, ifosfamide, and etoposide) or VAIA (vincristine, actinomycin D, ifosfamide, and doxorubicin) regimen. The expression levels of miR-125b were significantly upregulated in the resection samples consisting of viable tumor cells after chemotherapy (Figure 1C). The common upregulation of miR-125b in two different Dox-resistant cells, as well as in the clinical EWS tumor cells having survived chemotherapy, suggests that miR-125b may be involved in the acquisition of Dox-resistance in EWS cells.

**Figure 2:**
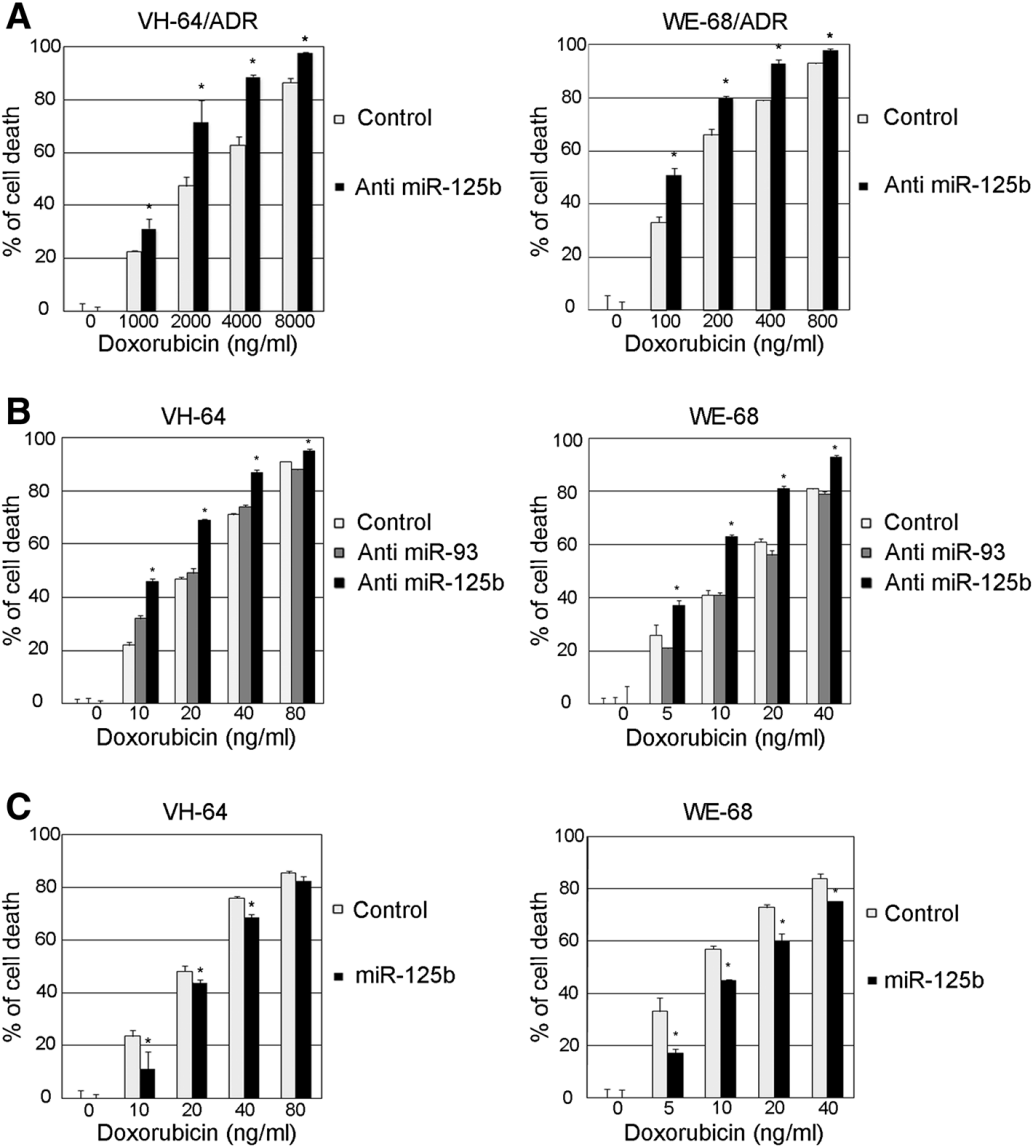
**The effects of miR-125b on the Dox-induced cytotoxicity in EWS cells.** An antisense (miR-125b and miR-93) or control construct was introduced into the Dox-resistant cells **A**), and the parental cells **B**) using a lentivirus. The cells were cultured with puromycin for 2 weeks to ensure a stable knockdown, and then were seeded at 2 × 10^3^ cells/well in 96 well plates. Twelve hours later, the cells were treated with various concentrations of doxorubicin for an additional 48 h. The cell viability was detected by the CellTiter-GloTM Luminescent Cell Viability Assay. The data represent the means of three separate experiments. The results are the means ± SD. *, P < 0.05. **C**) Cells were seeded at 2 × 10^3^ cell/well in 96 well plates 36 h after transfection with 100 nM control or has-miR-125b miRNA Precursor. The cells were treated with various concentrations of doxorubicin for an additional 48 h. The cell viability was detected by the CellTiter-GloTM Luminescent Cell Viability Assay. The data represent the means of three separate experiments. The results are the means ± SD. *, P < 0.05.

We next examined whether miR-125b could modulate the chemosensitivity to doxorubicin in EWS cells. When miR-125b was knocked down in the Dox-resistant cell lines, significantly enhanced cell death was observed (Figure 2A). The IC50 value shifted from 2570 to 1480 ng/ml in the VH-64/ADR cells, and from 188 to 96 ng/ml in the WE-68/ADR cells. The increased sensitivity to doxorubicin was also observed in parental cells after downregulating miR-125b (Figure 2B, from 21.8 ng/ml to 12.0 ng/ml in VH-64 cells, and from 13.6 to 7.0 ng/ml in WE-68 cells). The enhanced sensitivity to doxorubicin induced by downregulating miR-125b was also observed in three other EWS cell lines, RD-ES, SK-ES, and TC-71 (Additional file 1: Figure S1). To demonstrate the effect of miR-125b was not off-target, we also knocked down miR-93 which was about 1.5 times upregulated in Dox-resistance cells (Figure 1A). Down-regulating miR-93 induced no significant changes in the chemosensitivity to doxorubicin in EWS cells (Figure 2B). In contrast, when miR125b was overexpressed in the parental cells, significantly less cell death was induced by doxorubicin (Figure 2C). The IC50 values shifted from 21.8 to 26.7 ng/ml in the VH-64 cells, and from 8.1 to 12.6 ng/ml in the WE-68 cells. These results indicate that miR-125b enhances the chemoresistance of EWS cells to doxorubicin.

### miR-125b downregulates the pro-apoptotic molecules, p53 and Bak

**Figure 3:**
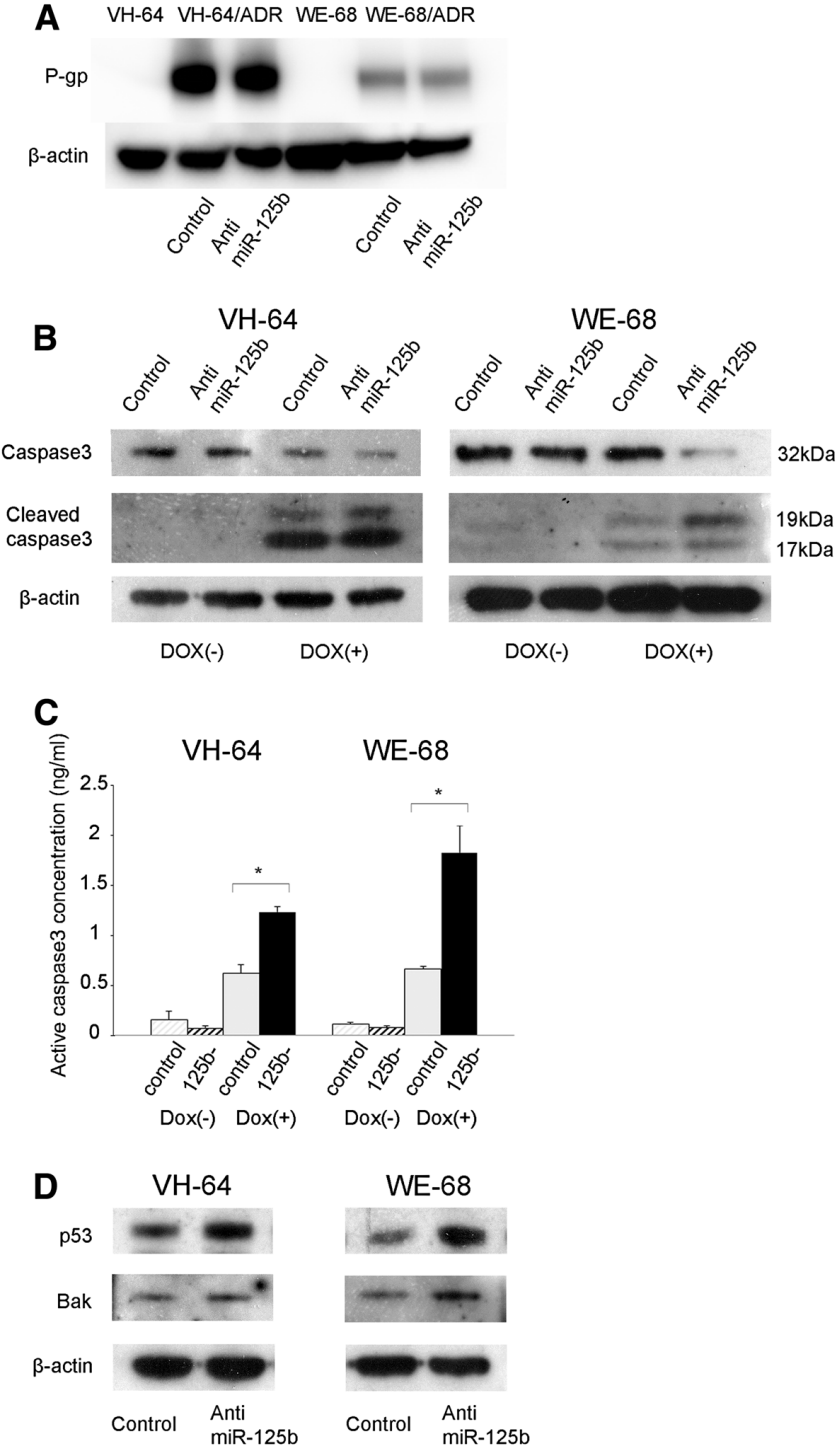
**Suppression of the expression of p53 and Bak by miR-125b. A**) miR-125b was stably knocked down in Dox-resistant EWS cells, and the whole cell lysates were subjected to an immunoblot analysis using antibodies against P-gp and β-actin. **B**), **C**) miR-125b was stably knocked down in EWS cells, and the cells were incubated with or without 100 ng/ml of doxorubicin for 24 h. The whole cell lysates were subjected to an immunoblot analysis using antibodies against caspase 3, cleaved caspase 3, and β-actin (**B**). The active caspase 3 levels were quantitated by using ELISA (**C**). **D**) miR-125b was stably knocked down in EWS cells, and the whole cell lysates were subjected to an immunoblot analysis using antibodies against p53, Bak, and β-actin.

We have previously reported that drug-efflux pump was overexpressed in Dox-resistance cells and developed chemoresisitance [16]. An immunoblot assay revealed no changes in P-gp in the Dox-resistant cells after down-regulating miR-125b (Figure 3A). Since miR-125b can affect chemosensitivity in the parental cells (Figure 2B) where efflux pumps were almost undetectable (Figure 3A) [16], additional mechanisms are likely to be involved in miR-125b-related chemosensitivity. In addition to drug efflux pumps, the induction of apoptosis is one of the most important mechanisms by which various anti-cancer drugs, including doxorubicin, exert their therapeutic effects [21]. Therefore, we investigated whether miR-125b can modulate apoptosis. When parental EWS cells were treated with doxorubicin, cleavage of caspase 3 was induced, indicating the induction of apoptotic cell death (Figure 3B). The enhanced cleavage of caspase 3 was quantitatively confirmed by ELISA (Figure 3C). When miR-125b was stably downregulated, enhanced cleavage of caspase 3, along with enhanced cell death (Figure 2B), was induced upon doxorubicin treatment (Figures 3B, C). This suggests that miR-125b may modulate chemoresistance by regulating apoptosis.

To elucidate the mechanism underlying how miR-125b regulates apoptosis, we sought the target gene(s) of miR-125b by using the web-based prediction program, TargetScan (http://www.targetscan.org/). Among the various predicted target genes, we focused on p53 and Bak, because they are involved in apoptotic signaling. In addition, both p53 and Bak have been reported to be direct targets of miR-125b in other types of cancer [22-25]. An immunoblot assay revealed that the expression levels of p53 and Bak were upregulated when miR-125b was stably knocked down in the parental EWS cells (Figure 3D), indicating that miR-125b suppressed these apoptotic mediators.

To validate the binding of miR-125b to the 3′UTR of p53 and Bak, we performed a luciferase reporter assay. Consistent with the findings of previous reports [22-25], the ectopic expression of miR-125b suppressed the activity of luciferase construct containing the 3′UTR of p53 and Bak (Additional file 2: Figure S2). These observations indicate that miR-125b directly targets p53 and Bak in EWS cells.

**Figure 4:**
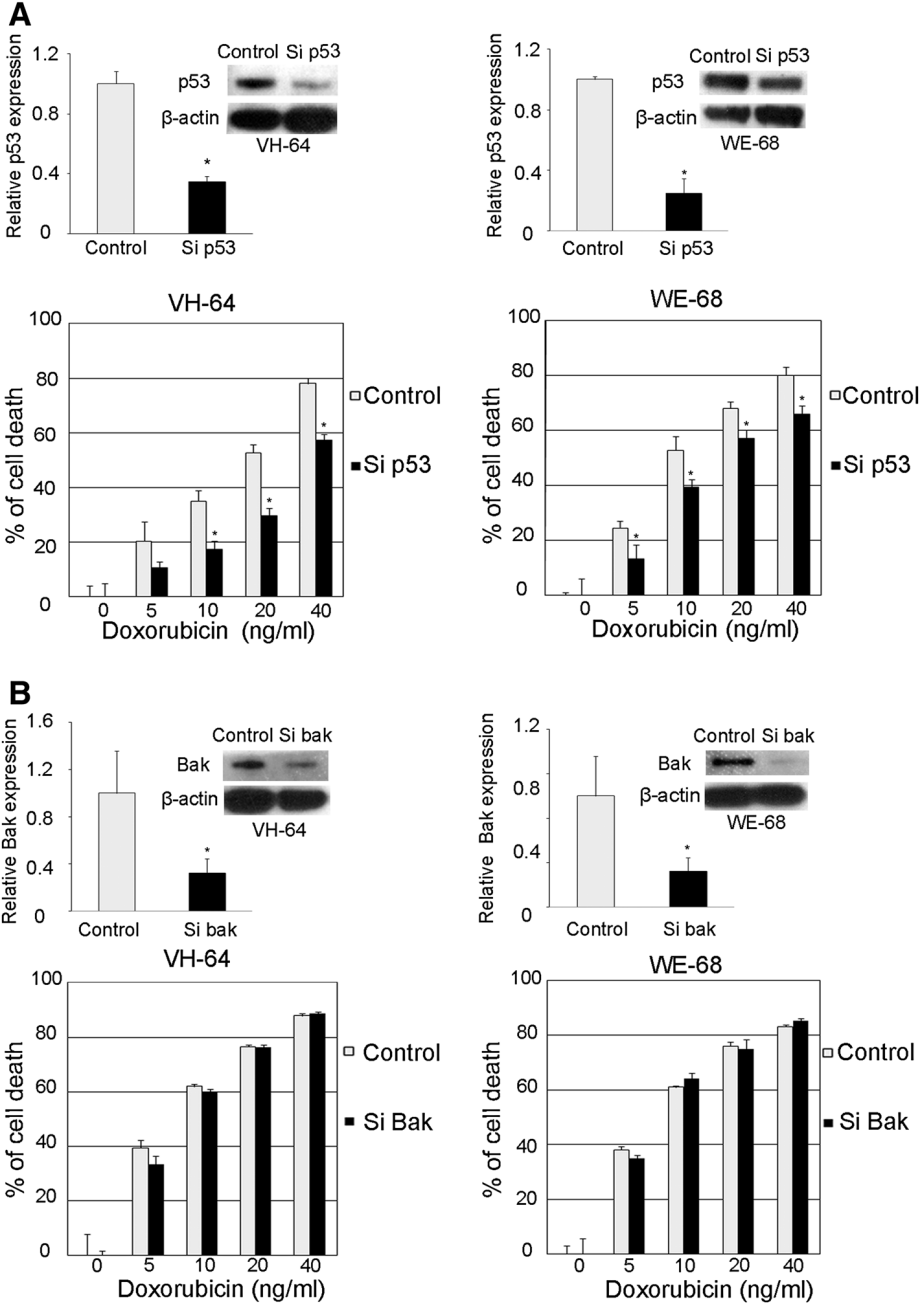
**Changes in Dox-induced cytotoxicity after p53 and Bak knockdown in EWS cells.** The VH-64 or WE-68 parental cell lines were transfected with 20 nM of siRNA against p53 (**A**) or Bak (**B**). After 48 h incubation, the cells were collected, and qRT-PCR and an immunoblot analysis were performed to confirm the effects of the siRNA. The cells were seeded at 2 × 10^3^ cells/well in 96 well plates and treated with various concentrations of doxorubicin for 48 h. The cell viability was determined by the CellTiter-GloTM Luminescent Cell Viability Assay. The data represent the means of three separate experiments. The results are the means ± SD. *, P < 0.05.

We then examined the roles of p53 and Bak in Dox-induced cell death. The p53 protein has been reported to sensitize EWS Rh1 cells to doxorubicin [14]. Bak is a relatively novel pro-apoptotic gene of the Bcl-2 family, and is known to mediate Dox-induced apoptosis in myeloma and lymphoma cell lines [26]. When p53 was knocked-down, both the VH-64 and WE-68 parental cells showed significant resistance to Dox-treatment (Figure 4A). The IC50 values shifted from 17.9 to 33.3 ng/ml in the VH-64 cells, and from 9.4 to 16.7 ng/ml in the WE-68 cells. In contrast, when Bak was knocked down in either VH-64 or WE-68 cells, no significant changes were observed in the Dox-related cytotoxicity (Figure 4B).

**Figure 5:**
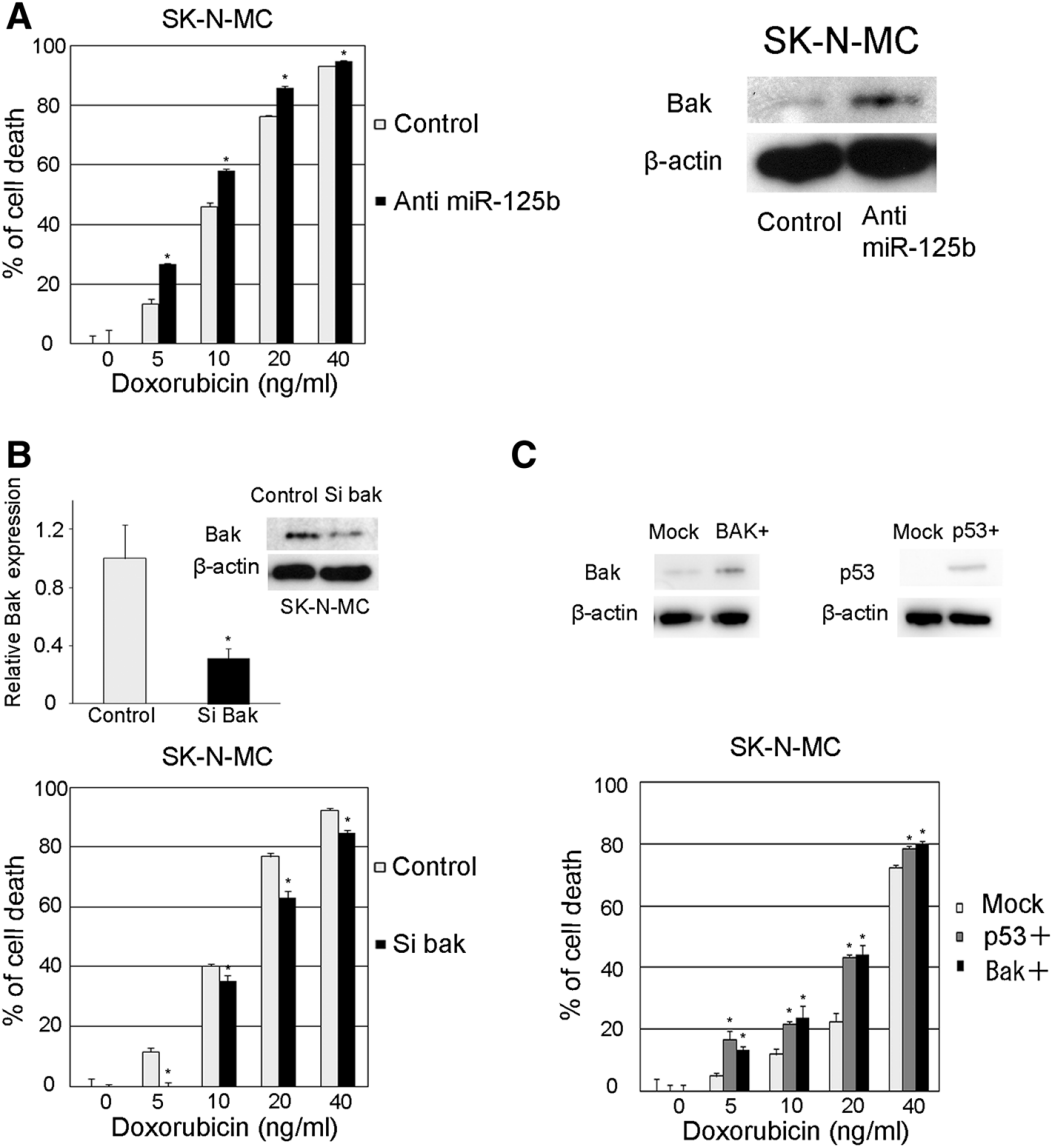
**Changes in Dox-induced cytotoxicity after the downregulation of miR-125b and Bak in p53-truncated EWS cells. A**) miR-125b was stably knocked down in SK-N-MC cells which express a truncated mutant of p53. The cells were seeded at 2 × 10^3^ cells/well in 96 well plates and cultured for 12 h, then were treated with various concentrations of doxorubicin for an additional 48 h. (Left) The cell viability was determined by the CellTiter-GloTM Luminescent Cell Viability Assay. The data represent the means of three separate experiments. The results are the means ± SD. *, P < 0.05. (Right) The whole cell lysates were subjected to an immunoblot analysis using antibodies against Bak and β-actin. **B**) SK-N-MC cells were transfected with 20 nM of siRNA against Bak. (Upper) After 48 h incubation, the cells were collected, and qRT-PCR and an immunoblot analysis were performed to confirm the effect of the siRNA. (Lower) Cells were seeded at 2 × 10^3^ cells/well in 96 well plates, and were treated with various concentrations of doxorubicin for 48 h. The cell viability was determined by the CellTiter-GloTM Luminescent Cell Viability Assay. The data represent the means of three separate experiments. The results are the means ± SD. *, P < 0.05. **C**) p53, Bak or mock construct was introduced into SK-N-MC cells using a lentivirus. After 96 h incubation, the cells were seeded at 2 × 10^3^ cells/well in 96 well plates. Twelve hours later, the cells were treated with various concentrations of doxorubicin for an additional 48 h. The cell viability was detected by the CellTiter-GloTM Luminescent Cell Viability Assay. The data represent the means of three separate experiments. The results are the means ± SD. *, P < 0.05. Immunoblot analyses were performed to confirm the effects of the lentivirus induction.

We were interested in determining whether miR-125b could modulate the chemosensitivity in an EWS cell line expressing a truncated p53 mutant, SK-N-MC [27]. When miR-125b was knocked down in the cell, significantly enhanced cell death was observed upon Dox-treatment (the IC50 value shifted from 11.0 to 8.4 ng/ml), and the expression level of Bak was significantly enhanced (Figure 5A). When Bak was knocked down in the SK-N-MC cell line, the cells showed significant resistance to Dox-treatment (Figure 5B). In contrast, when p53 and Bak were overexpressed, the cells showed significant sensitivity to doxorubicin (Figure 5C). These results suggest that miR-125b enhances chemoresistance by downregulating the p53/Bak apoptotic pathway in EWS cells.

### miR-125b regulates multidrug resistance in EWS cells

**Figure 6:**
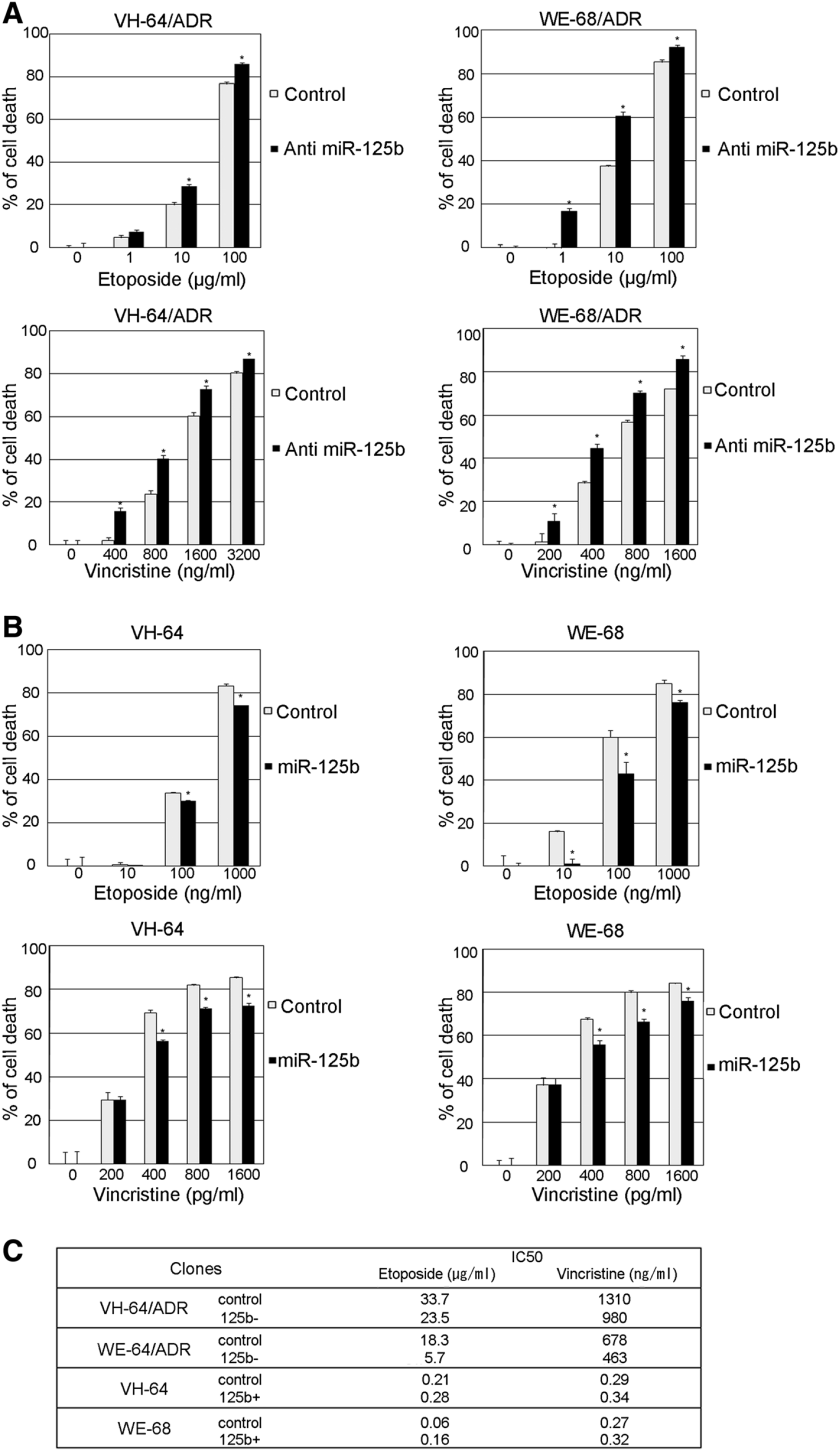
**The effects of miR-125b on the resistance of EWS cells to etoposide and vincristine. A**) miR-125b was stably knocked down in Dox-resistant EWS cells. The cells were seeded at 2 × 10^3^ cells/well in 96 well plates, cultured for 12 h, and then treated with various concentrations of vincristine or etoposide for an additional 48 h. The cell viability was determined by the CellTiter-GloTM Luminescent Cell Viability Assay The data represent the means of three separate experiments. The results are the means ± SD. *, P < 0.05. **B**) Cells were seeded at 2 × 10^3^ cells/well in 96 well plates 36 h after transfection with 100 nM control or has-miR-125b miRNA Precursor. The cells were treated with various concentrations of vincristine or etoposide for an additional 48 h. The cell viability was determined by the CellTiter-GloTM Luminescent Cell Viability Assay. The data represent the means of three separate experiments. The results are the means ± SD. *, P < 0.05. **C**) The drug sensitivity of the EWS clones to various anticancer agents. IC50 showed drug concentrations that inhibited cell survival by 50%.

We have previously reported that Dox-resistant EWS cells expressed P-gp and MRP1, and showed cross-resistance to histone deacetylase inhibitors [16]. Because the VDC-IE protocol has been accepted as a standard regimen for treating EWS [28], we wanted to determine whether miR-125b could modulate the sensitivity to the above drugs. Among the 5 drugs in the standard regimen, cyclophosphamide and ifosfamide were not investigated because they are prodrugs, and cannot be evaluated in *in vitro* studies. Instead, we used mafosfamide, a pre-activated cyclophosphamide analog, as a subsutitute for cyclophosphamide. As shown in Figure 6, Dox-resistant cells showed significant cross-resistance to vincristine and etoposide. The downregulation of miR-125b in the resistant cells increased their sensitivity to vincristine and etoposide (Figure 6A, C). In contrast, when miR-125b was overexpressed in the parental cells, reduced cytotoxicity was observed upon treatment with vincristine and etoposide (Figure 6B, C). Intriguingly, the Dox-resistant cells showed almost the same sensitivity to mafosfamide as the parental cells. Knocking down miR-125b enhanced the sensitivity of both parental and resistant cells to mafosfamide (Additional file 3: Figure S3). These observations suggest that miR-125b enhances the chemoresistance to multiple drugs used in the standard regimen for treating EWS.

## Discussion

One major mechanism of drug resistance in cancer cells is the evasion of apoptosis [7,29]. Recent findings have revealed that miRNAs can modulate drug resistance by impairing the apoptotic pathway in various cancers [19,24,30,31]. In this study, we observed the upregulation of miR-125b in Dox-resistant EWS cells. When miR-125b was knocked down in EWS cells, both the Dox-resistant cells and the Dox-sensitive parental cells showed enhanced chemosensitivity to doxorubicin, and this was associated with the upregulation of the pro-apoptotic molecules, p53 and Bak. Conversely, overexpressing miR-125b in EWS cells resulted in enhanced drug resistance. We have previously reported the involvement of ABC transporters during the acquisition of multidrug resistance in EWS cells [15,16]. However, the drug resistance in those cells was not fully reversed in the presence of efflux pump inhibitors, so it was concluded that additional mechanisms of resistance were also likely to be involved. Our present observations clearly revealed the involvement of miR-125b during the acquisition of multidrug resistance in EWS cells.

It is known that miR-125b is a vertebrate homologue of the C. elegans microRNA lin-4, which regulates the reiterations of stem cells in C. elegans [32]. Similar to lin-4, miR-125b has been shown to regulate the homeostasis of mammalian neural and hematopoietic stem cells [33,34]. Several targets of miR-125b have been identified, including ETS [35], ERBB [36], p53 [37], Bak [23,24], and Lin28 [34], thus suggesting the involvement of miR-125b and its targets in proliferation and apoptosis.

Recent reports suggest that miR-125b functions as a tumor suppressor in some types of tumors, such as breast cancer [35,38], thyroid cancer [39], and hepatocellular carcinoma [40]. In contrast, miR-125b was shown to function as an oncogene in B-cell leukemia [41], endometrial carcinoma [42], and colorectal cancer [43]. Shi et al. reported that the downregulation of miR-125b induced growth inhibition of prostate cancer cells, whereas the overexpression of miR-125b enhanced cell growth [23]. Our findings revealed that miR-125b acts as an oncogene in EWS cells by targeting p53 and Bak.

The tumor suppressor p53, recognized as the “guardian of the genome”, regulates many downstream genes and plays a pivotal role in regulating the cell cycle and cell death. Recent studies have revealed that several miRNAs, including miR-125b, directly target p53 [22]. The loss of miR-125b increases widespread p53-dependent apoptosis, leading to severe defects in zebrafish embryos [37]. Direct suppression of p53 by miR-125b affects the camptothecin-induced apoptosis in various cancer cell types [25], and was assumed to be associated with a poor prognosis of colorectal cancer [43]. The results of our current study are consistent with these previous reports that miR-125b acts as an oncogene by suppressing p53-dependent apoptosis (Figure 3). Intriguingly, an anti-apoptotic role of miR-125b, mediated through the suppression of multiple pro-apoptotic regulators in the p53 network, is conserved in vertebrates [37], suggesting the importance of miR-125b in regulating p53.

Although the function of p53 is reported to be disrupted in a wide variety of tumors, p53 mutations are uncommon in EWS. The majority of EWS tumors express wild-type p53 [44]. Instead of genetic alterations, the inactivation of p53 during the development and progression of EWS has been explained by the interaction between p53 and the EWS/FLI1 fusion gene [45]. We have reported that EWS/FLl1 interacts with p53, impairs its transcriptional activity, and inhibits the expression of its downstream target genes [46]. In addition to these post-translational modifications by EWS/FLI1, miR-125b may regulate the expression of p53 post-transcriptionally by interacting with its 3′UTR in EWS [22,37].

Bak is a pro-apoptotic mitochondrial membrane protein, usually inactivated by the formation of complexes with the anti-apoptotic Bcl2 family protein, Mcl1. Bak has been reported to be a target of miR-125b, which has been implicated in the androgen-independent growth of prostate cancer cells [23], and also in paclitaxel-induced apoptosis in breast cancer cells [47]. In this study, we showed that Bak is also involved in miR-125b-mediated Dox-resistance in EWS cells. The downregulation of Bak reduced chemosensitivity only in the p53-truncated SK-N-MC cells (Figure 5B), indicating the importance of wild-type p53 during Dox-induced cytotoxicity in EWS. Of note, p53 was shown to bind to Bak following genotoxic stress, and to induce its oligomerization, leading to cytochrome c release [48]. Although we have not examined the direct interaction of p53 with Bak in EWS cells, these observations indicate that, in the absence of p53, the miR-125b-Bak axis plays a role in the chemosensitivity in EWS cells.

The role of microRNAs in EWS remains largely unclear. Very recently, Italian investigators revealed the involvement of miR-34a in the chemoresistance of EWS [20]. They screened miRNAs by discriminating EWS patients with different clinical outcomes and successfully identified miR-34a as a regulator of chemosensitivity and a possible prognostic marker. On the other hand, we screened Dox-resistant EWS cells and found miR-125b to be upregulated in the resistant cells. Upregulation of miR-125b was also confirmed in the EWS tumors having survived chemotherapy regimens that included doxorubicin. Although the screening methods are different in these studies, both studies clearly demonstrate the involvement of miRNAs in the development of chemoresistance in EWS.

Upregulation of miR-125b has been reported in various tumors, including B-cell leukemia, endometrial carcinoma and colorectal cancer [41-43]. In regard to Dox-resistant cell lines, upregulation of miR-125b was observed in the malignant peripheral nerve sheath tumor cells, FU-SFT9817, but not in the osteosarcoma cell line MNNG (Additional file 4: Figure S4). It appears that the upregulation of miR-125b in tumor cells occurs in a cell type-dependent manner. Thus far, the underlying mechanism responsible for this upregulation remains unclear. The contribution of NF-kB p65 binding sites as well as CpG-rich regions upstream of miR-125b-1 in the regulation of miR-125b has been postulated [35,47]. We observed no differences in the copy numbers of miR-125b genes between the parental and Dox-resistant EWS cells (Additional file 5: Figure S5), suggesting epigenetic regulation of the miR-125b expression in Dox-resistant EWS cells. Further studies are required to elucidate the mechanisms regulating the miR-125b expression.

Since the introduction of the VDC-IE regimen, the 5-year survival rates for patients with localized disease have ranged from 60 to 70% [1,28]. Nevertheless, EWS still has a low survival rate because of the frequent development of recurrence and/or metastatic lesions, which are usually associated with the acquisition of multidrug resistance [28]. We have observed that miR-125b significantly affected the chemosensitivity of EWS to doxorubicin, vincristine, etoposide (Figure 6), and mafosfamide (Additional file 3: Figure S3). As shown in Figure 1C, miR-125b was significantly upregulated in EWS tumors after VDC-IE or VAIA treatment. Upregulation of miR-125b upon chemotherapy have been reported in colorectal cancer [49], and breast cancer [47]. These observations suggest that the acquisition of drug resistance may be regulated, at least partly, via the miR-125b-p53/Bak pathway.

## Conclusions

In summary, miR-125b was commonly upregulated in Dox-resistant EWS cells as well as in EWS tumors having survived chemotherapy. miR-125b led to the development of chemoresistance by suppressing the expression of p53 and Bak, and repression of miR-125b sensitized EWS cells to apoptosis induced by treatment with various cytotoxic drugs. Elucidating the involvement of miRNAs in the development of chemoresistance should be required to further improve the clinical prognosis for EWS.

## Methods

### Reagents

Doxorubicin was obtained from Kyowa Hakko (Tokyo, Japan). Etoposide was obtained from Calbiochem (San Diego, CA). Vincristine and mafosfamide were obtained from Wako (Osaka, Japan).

### Cells and cell culture

The human EWS cell lines, VH-64 and WE-68 (with wild type p53), and SK-N-MC (with a p53 truncation), and RD-ES, SK-ES, and TC-71 (with mutant type p53) were cultured in RPMI-1640 (Invitrogen, Carlsbad, CA) containing 10% fetal bovine serum (HyClone Laboratories, Inc., Logan, UT). The Dox-resistant EWS clones VH-64/ADR and WE-68/ADR were established and characterized in our laboratory [16], and cultured in RPMI-1640 containing 10% fetal bovine serum with 100 ng/ml of doxorubicin. The cells were incubated at 37°C in a humidified atmosphere containing 5% CO_2_.

### miRNA extraction from clinical samples

The study population consisted of 11 serial cases retrieved from the archives of the Department of Anatomic Pathology, Pathological Sciences, Graduate School of Medical Science, Kyushu University, Japan. The tissue specimens were collected during primary tumor open biopsy at diagnosis between 2002 and 2008. In each case, a diagnosis of EWS was made based on the histological features of the specimen. From these 11 cases, six cases were excluded because of a lack of availability of adequate tissue. The remaining five cases were treated with systemic VDC-IE or VAIA regimen [1], and then the tumors were resected.

The expression profiles of miRNAs were reported to be in good correlation between fresh frozen and formalin-fixed, paraffin-embedded (FFPE) samples [50,51]. Therefore, all samples were prepared as FFPE sections. Thereafter, 4 μm sections of FFPE tissues were deparaffinized with xylene, washed in ethanol, and digested with proteinase K. Total RNA was extracted using a miRNeasy FFPE kit (Qiagen, Valencia, CA). Ten nanograms of total RNA from each sample were used for cDNA synthesis. The Institutional Review Board at Kyushu University approved the use of human specimens for this study (Reference number 21-124).

### Quantitative real-time PCR (qRT-PCR)

For the miRNA expression analysis, total RNA was purified from harvested cells using a miRNeasy Mini Kit (Qiagen). The total RNA was used in a reverse transcription reaction with a MiScript Reverse Transcription Kit (Qiagen). Real-time PCR was carried out using a LightCycler 1.5 (Roche Diagnotics, Indianapolis, IN) with miRNA-specific primers (Qiagen) according to the manufacturer’s instructions (miScript SYBR Green PCR kit, Qiagen). RNU6B was used as an internal control. The RT reactions were performed using the following conditions: an initial denaturation step at 95°C for 15 min, followed by 40 cycles of denaturation at 94°C for 15 s, annealing at 55°C for 30 s, and extension at 70°C for 30 s.

For the mRNA expression analysis [52], total RNA was extracted using an RNeasy kit (Qiagen). The total RNA was used in a reverse transcription reaction with SuperScriptII reverse transcriptase (Invitrogen). Real-time PCR was carried out using a LightCycler 1.5 with mRNA-specific primers according to the manufacturer’s instructions (Perfect Real Time, Takara Bio, Shiga, Japan). Glyceraldehyde 3-phosphate dehydrogenase (GAPDH) was used as an internal control. The RT reactions were performed under the following conditions: an initial denaturation step at 95°C for 5 s, followed by 40 cycles of denaturation at 95°C for 10 s, and annealing at 60°C for 30 s. The following primers were used: Bak (forward: 5′-CTTCGTGGTCGACTTCATGCT-3′ and reverse: 5′-GGACCATTGCCCAAGTTCAG-3′; product size 93 bp), p53 (forward: 5′-ACTAAGCGAGCACTGCCCAAC-3′ and reverse: 5′-CCTCATTCAGCTCTCGGAACATC-3′; product size 130 bp), and GAPDH (forward: 5′-GAAGGT GAAGGTCGGAGTC-3′ and reverse: 5′-GAAGATGGT GATGGGATTTC-3′; product size 226 bp).

The expression levels of miRNAs, mRNAs, and DNAs were calculated using the LightCycler version 3.5 software program (Roche Diagnotics). A negative control was also prepared using distilled water instead of a DNA template. The assay was performed in triplicate and was repeated in at least three separate experiments.

### siRNA and miRNA experiments

The cells were seeded at 1.5 × 10^5^ cells per well in 6 well plates. After 24 h in culture, the cells were transfected with p53 siRNA (Ambion ID 605), Bak siRNA (Ambion ID 1880), has-miR-125b miRNA Precursor (Ambion ID PM10148) or Silencer Negative Control #1 siRNA (Ambion, Austin, TX) using Lipofectamine2000 (Invitrogen) according to the manufacturer’s protocols. The introduction of the miRNAs was confirmed by qRT-PCR (Additional file 6: Figure S6), and the introduction of the siRNAs was confirmed by qRT-PCR and immunoblotting. Thirty-six hours after transfection, a chemosensitivity assay was performed as described below.

### Chemosensitivity assay

For the chemosensitivity assay, cells were seeded at 2 × 10^3^ cells per well in 96 well plates. After 12 h incubation, various concentrations of drugs were added to the medium. After another incubation for 48 h, the number of viable cells in each well was measured using the CellTiter-GloTM Luminescent Cell Viability kit (Promega, Madison, WI), according to the manufacturer’s protocol. Before the use of Dox-resistant clones in this assay, they were cultured in medium without doxorubicin for 10 days. The chemosensitivity assay was carried out in triplicate and was repeated at least three times in separate experiments.

### Profiling microRNA expression by Luminex

The total RNA from VH-64 and VH-64/ADR cells was extracted using a Vantage™ Total RNA Purification Kit (Origene, Rockville, MD). A 2 μg aliquot of total RNA was labeled with biotin by the Vantage™ microRNA Labeling Kit (Origene) as described in the manufacturer’s protocol. The labeled total RNA was hybridized to Bead Mix (Vantage™ microRNA Multiplex Detection Kit Oncology Detection Panel, Origene) at 60°C by using a thermocycler for one hour. The hybridized reactions were transferred to 96 well filter plates. After several washing steps, the filter plate was read using a Luminex100 instrument (Luminex Corp., Austin, TX) [53].

### Lentiviral vector construction and production

miRNA expression vectors (miRZip Anti-miR-125b microRNA Construct, miRZip Anti-miR-93 microRNA Construct and Scramble Hairpin Control Anti-microRNA Construct, System Biosciences, Mountain View, CA) and the Lentivirus Package plasmid mix (System Biosciences) were co-transfected into 293TN (System Biosciences) cells with Lipofectamine2000 (Invitrogen) according to the manufacturer’s protocol. The culture supernatants were collected 48 h post-transfection. The supernatant was concentrated by PEG-it Virus Precipitation Solution (System Biosciences), and the concentrated supernatant was used to infect target cells.

To create p53 and Bak lentiviral vectors, we inserted p53 and Bak cDNA into pCDH-EF1-MCS-IRES-Puro (System Biosciences). These vectors were co-transfected with Package plasmid mix into 293TN, and the concentrated supernatant was used.

### Lentiviral infection

Cells were plated at a density of 0.5 × 10^5^ cells per well in 24 well plates with RPMI (with serum, but without antibodies). After 24 h incubation, the cells were transfected with lentivirus particles using the TransDux Virus Transduction Reagent (System Biosciences). Two days after transfection, puromycin was added to the medium to establish cells with stable miR-125b knock down. Almost all cells were confirmed to be infected by counting GFP-positive cells 2 weeks after the transfection (Additional file 7: Figure S7).

### Western blot analysis

The cells were washed twice with ice-cold PBS, scraped, collected in a microcentrifuge tube, and then centrifuged. The cells were lysed using CelLytic (Sigma Aldrich, St. Louis, MO) with a protease inhibitor cocktail (Complete Mini, EDTA-free; Roche Diagnotics). After incubating the cells for 10 min on ice, the cellular debris was pelleted by centrifuging for 15 min at 12000 × g at 4°C. The protein quantity in the lysate was determined using a Bradford protein assay (Bio-Rad, Richmond, CA). The samples were boiled for 5 min, and each of the samples was separated on a 4–12% gradient pre-cast MOPS polyacrylamide gel (Novex, San Diego, CA) and transferred onto a nitrocellulose membrane. The filter was blocked with TBS containing 5% non-fat dry milk and 0.1% Tween20 for 1 h at room temperature. The filter was then incubated overnight with the appropriate primary antibodies at 4°C. The following primary antibodies were used: P-gp (Alexis Biochemicals, San Diego, CA), p53 and β-actin (Santa Cruz Biotechnology, Santa Cruz, CA), Bak (Abcam, Cambridge, UK), caspase 3 (BD Biosciences, San Jose, CA), and cleaved caspase 3 (Cell Signaling, Beverly, MA). After washing the filter, a horseradish peroxidase-conjugated secondary antibody (Santa Cruz Biotechnology) was added, and the filter was incubated at room temperature for 1 h. After a final wash with TBST, the immunoreactivity of the blots was detected using an enhanced chemiluminescence (ECL) detection system (Amersham, Buckinghamshire, UK).

### Active caspase 3 ELISA

Cells were seeded at 5 × 10^5^ cells per well in 12 well plates and incubated with 100 ng/ml doxorubicin. After 24 h incubation, the cells were harvested. The concentrations of active caspase 3 were measured according to the manufactures instructions (R&D Systems, Minneapolis, MN).

### Statistical analysis

Statistical comparisons were performed using Student’s *t-*test. The minimal level of significance was considered to be P = 0.05.

## Consent

Written informed consent was obtained from the patient for publication of this report and any accompanying images.

## Abbreviations

miRNA: microRNA; EWS: Ewing sarcoma/primitive neuroectodermal tumor; Dox: Doxorubicin; P-gp: P-glycoprotein; GAPDH: Glyceraldehyde 3-phosphate dehydrogenase; FFPE: Formalin-fixed, paraffin-embedded

## Competing interests

The authors declare that they have no competing interests.

## Authors’ contributions

KI, JF, YI designed research and analyzed data. YM, YO, YT, TF, YF-O, MH, AN, and SK carried out molecular biology studies. KI and JF wrote the paper. All authors read and approved the final manuscript.

## Supplementary Material

Additional file 1: Figure S1 The effects of miR-125b on the Dox-induced cytotoxicity in EWS cells. miR-125b was stably knocked down in EWS cells (RD-ES, SK-ES, and TC-71). The cells were seeded at 2 × 10^3^ cells/well in 96 well plates, cultured for 12 h, and then treated with various concentrations of doxorubicin for an additional 48 h. The cell viability was determined by the CellTiter-GloTM Luminescent Cell Viability Assay The data represent the means of three separate experiments. The results are the means ± SD. *, P < 0.05. Click here for file

Additional file 2: Figure S2 3′UTR-luciferase assay for p53 and Bak. Reporter luciferase vectors containing the 3′UTR of p53 and Bak were purchased from Ambion, and random control vectors were purchased from Switchgear genomics (Menlo Park, CA). Cells were seeded at a density of 2 × 10^5^ cells per well in 12 well plates. The cells were co-transfected with luciferase reporters and has-miR-125b miRNA Precursor or Silencer Negative Control #1 siRNA. After 36 h incubation, the cells were collected. The luciferase activity was measured using a dual luciferase reporter assay (Promega). The pRL-TK vector was used as an internal control. The results are expressed as the relative luciferase activity (firefly Luc/Renilla Luc). The results are the means ± SD. *, P < 0.05. Click here for file

Additional file 3: Figure S3 The effects of miR-125b on the sensitivity of EWS cells to mafosfamide. An antisense or control construct was introduced into the parental cells (A), and the Dox-resistant cells (B) using a lentivirus. The cells were cultured with puromycin for 2 weeks to ensure a stable knockdown, and then were seeded at 2 × 10^3^ cells/well in 96 well plates. Twelve hours later, the cells were treated with various concentrations of mafosfamide for an additional 48 h. The cell viability was detected by the CellTiter-GloTM Luminescent Cell Viability Assay. The data represent the means of three separate experiments. The results are the means ± SD. *, P < 0.05. Click here for file

Additional file 4: Figure S4 The miRNA expression levels in the Dox-resistant tumor cells. qRT-PCR was performed to investigate the expression of miR-125b in Dox-resistant cell lines. RNU6B was used as an internal control. The data represent the means of three separate experiments. The results are the means ± SD. *, P < 0.05. Click here for file

Additional file 5: Figure S5 The copy number analysis of miR-125b in VH-64 and VH-64/ADR. Quantitative-PCR was performed to examine the copy numbers of miR-125b in VH-64 and VH-64/ADR. DNA was extracted using DNeasy (Qiagen), and real-time PCR was carried out using a LightCycler 1.5 with specific primers according to the manufacturer’s instructions (Perfect Real Time, Takara Bio). The GAPDH and TNSALP (Tissue non-specific alkaline phosphatase) genes were used as control 1 and control 2, respectively. The PCR reactions were performed under the following conditions: an initial denaturation step at 95°C for 5 s, followed by 40 cycles of denaturation at 95°C for 10 s and annealing at 60°C for 30 s. The following primers were used: miR-125b-1 (forward: 5′- CTGGT CACCTGATCCCATCT-;3′ and reverse: 5′-ATTGTTGCGCTCCTCTCAGT-3′; product size 217 bp), miR-125b-2 (forward: 5′-CCGCATCAAACCAGACT TTT-3′ and reverse: 5′-GGATGGGTCATGGTGAAAAC-3′; product size 228 bp), control 1 (forward: 5′-CAACGAATTTGGCTACAGCA-3′ and reverse: 5′- AGGGGTCTACATGGCAACTG-3′; product size 195 bp), and control 2 (forward: 5′- AGGAGCACGAGAGACTGAGG-3′ and reverse: 5′- CTGGCT GCTGTCATGTTCAG-3′; product size 232 bp). The data represent the means of three separate experiments. Click here for file

Additional file 6: Figure S6 The induction of miR-125b in parental EWS cells. The VH-64 or WE-68 parental cell lines were transfected with100 nM control or has-miR-125b miRNA Precursor. After 48 h incubation, the cells were collected, and qRT-PCR was performed to confirm the expression of miR-125b. Click here for file

Additional file 7: Figure S7 The knockdown of miR-125b using the lentivirus. Cells were infected with the miRZip lentivirus construct, then the induction of the anti-miR-125b was confirmed by GFP-positivity in almost all of the cells. Click here for file
