# Supplementary figures and images for: miR-125b develops chemoresistance in Ewing sarcoma/primitive neuroectodermal tumor
Source: Cancer Cell Int. 2013 Mar 4;13:21. doi: 10.1186/1475-2867-13-21 (PMC3599506; doi:10.1186/1475-2867-13-21)

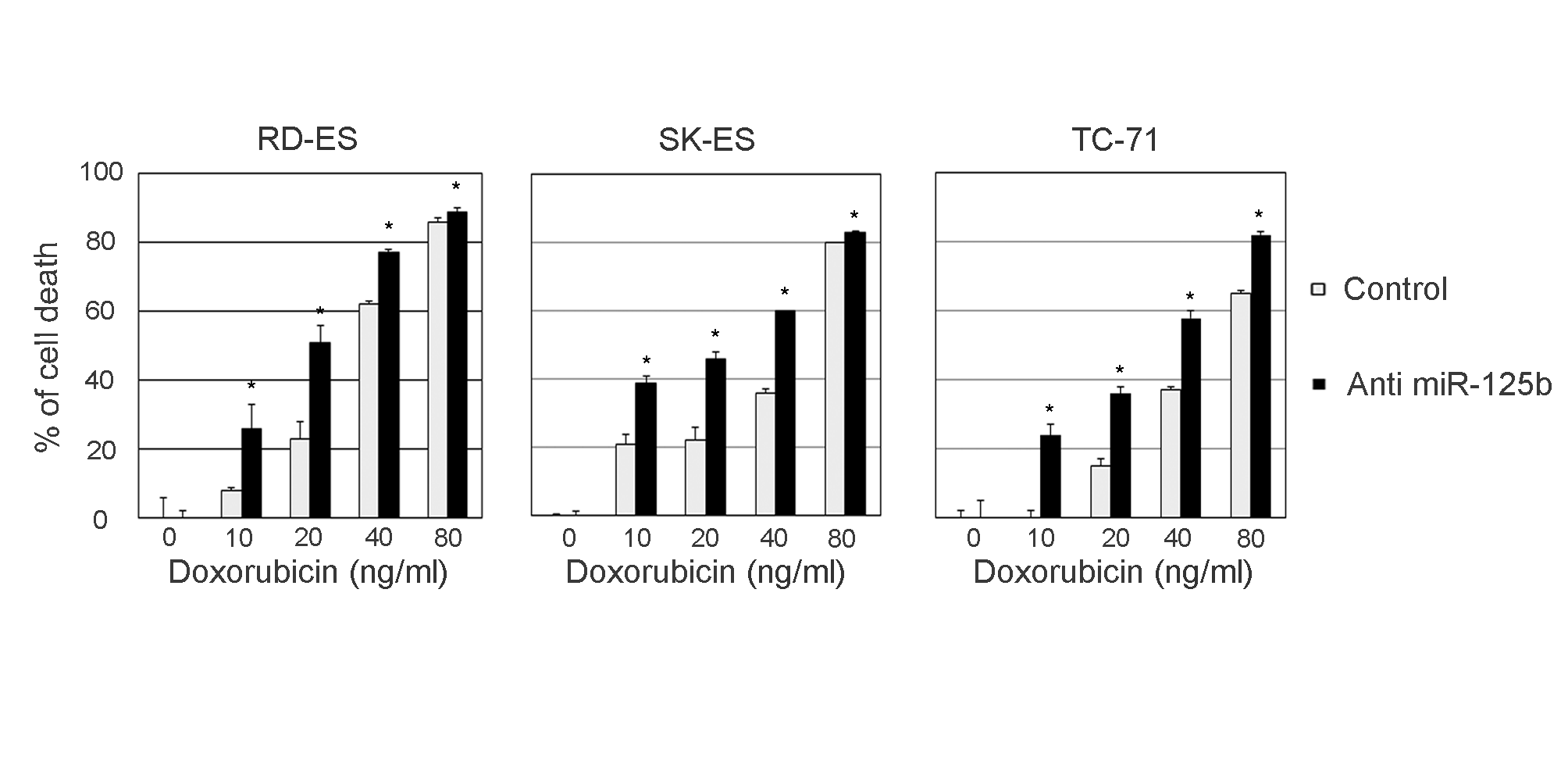

Supplement: Additional file 1: Figure S1 — The effects of miR-125b on the Dox-induced cytotoxicity in EWS cells. miR-125b was stably knocked down in EWS cells (RD-ES, SK-ES, and TC-71). The cells were seeded at 2 × 10^3^ cells/well in 96 well plates, cultured for 12 h, and then treated with various concentrations of doxorubicin for an additional 48 h. The cell viability was determined by the CellTiter-GloTM Luminescent Cell Viability Assay The data represent the means of three separate experiments. The results are the means ± SD. *, P < 0.05. [file 1475-2867-13-21-S1.tiff]

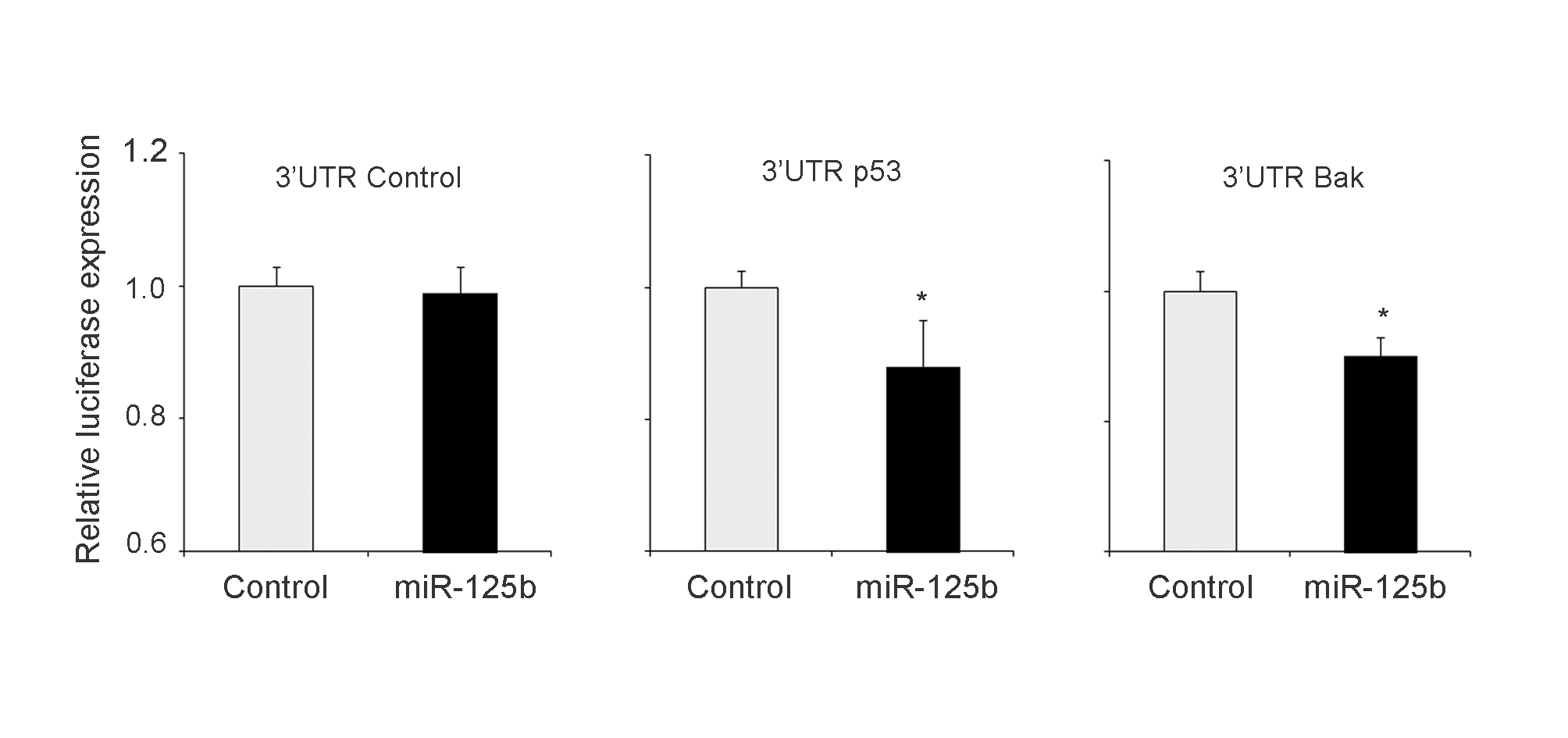

Supplement: Additional file 2: Figure S2 — 3′UTR-luciferase assay for p53 and Bak. Reporter luciferase vectors containing the 3′UTR of p53 and Bak were purchased from Ambion, and random control vectors were purchased from Switchgear genomics (Menlo Park, CA). Cells were seeded at a density of 2 × 10^5^ cells per well in 12 well plates. The cells were co-transfected with luciferase reporters and has-miR-125b miRNA Precursor or Silencer Negative Control #1 siRNA. After 36 h incubation, the cells were collected. The luciferase activity was measured using a dual luciferase reporter assay (Promega). The pRL-TK vector was used as an internal control. The results are expressed as the relative luciferase activity (firefly Luc/Renilla Luc). The results are the means ± SD. *, P < 0.05. [file 1475-2867-13-21-S2.tiff]

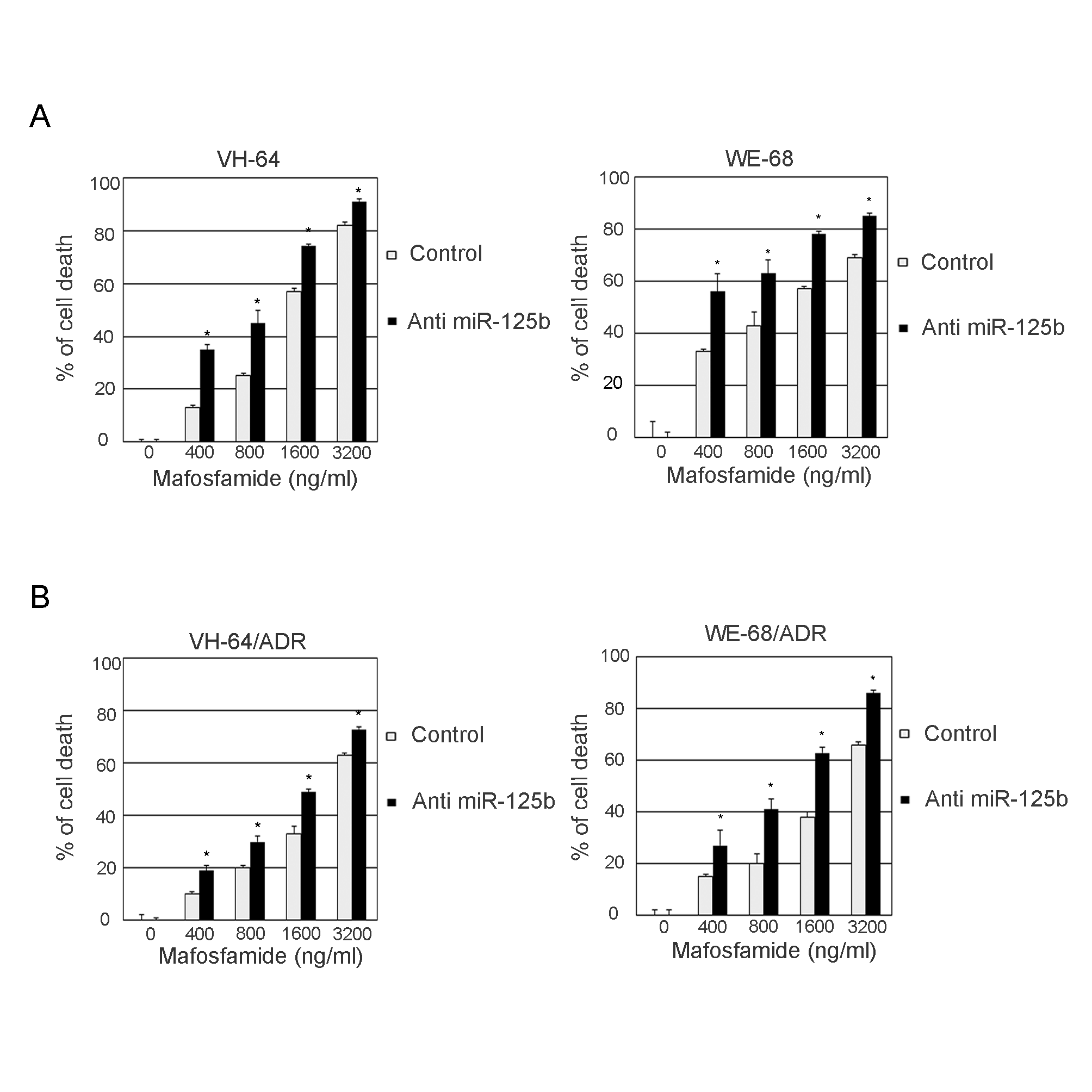

Supplement: Additional file 3: Figure S3 — The effects of miR-125b on the sensitivity of EWS cells to mafosfamide. An antisense or control construct was introduced into the parental cells (A), and the Dox-resistant cells (B) using a lentivirus. The cells were cultured with puromycin for 2 weeks to ensure a stable knockdown, and then were seeded at 2 × 10^3^ cells/well in 96 well plates. Twelve hours later, the cells were treated with various concentrations of mafosfamide for an additional 48 h. The cell viability was detected by the CellTiter-GloTM Luminescent Cell Viability Assay. The data represent the means of three separate experiments. The results are the means ± SD. *, P < 0.05. [file 1475-2867-13-21-S3.tiff]

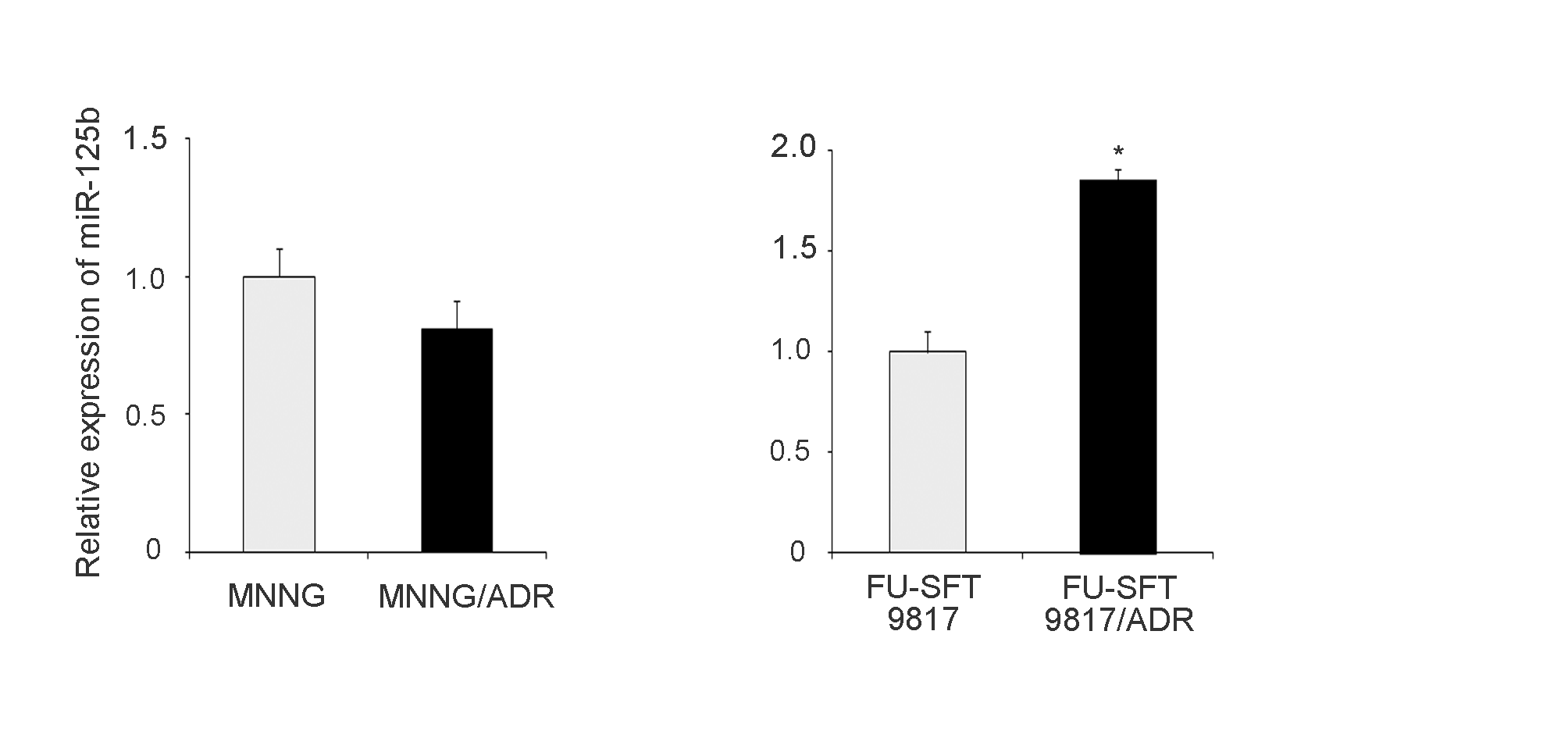

Supplement: Additional file 4: Figure S4 — The miRNA expression levels in the Dox-resistant tumor cells. qRT-PCR was performed to investigate the expression of miR-125b in Dox-resistant cell lines. RNU6B was used as an internal control. The data represent the means of three separate experiments. The results are the means ± SD. *, P < 0.05. [file 1475-2867-13-21-S4.tiff]

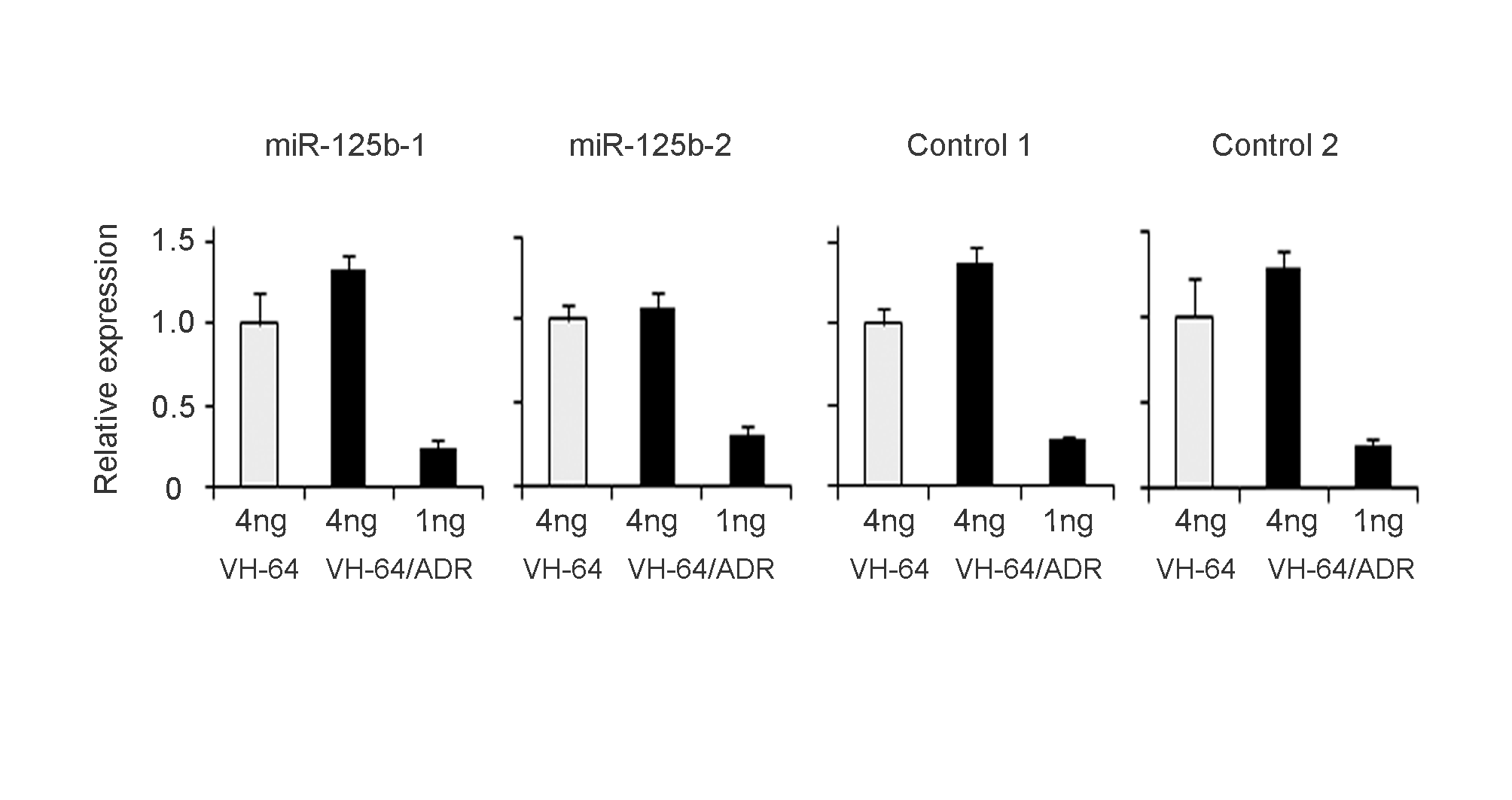

Supplement: Additional file 5: Figure S5 — The copy number analysis of miR-125b in VH-64 and VH-64/ADR. Quantitative-PCR was performed to examine the copy numbers of miR-125b in VH-64 and VH-64/ADR. DNA was extracted using DNeasy (Qiagen), and real-time PCR was carried out using a LightCycler 1.5 with specific primers according to the manufacturer’s instructions (Perfect Real Time, Takara Bio). The GAPDH and TNSALP (Tissue non-specific alkaline phosphatase) genes were used as control 1 and control 2, respectively. The PCR reactions were performed under the following conditions: an initial denaturation step at 95°C for 5 s, followed by 40 cycles of denaturation at 95°C for 10 s and annealing at 60°C for 30 s. The following primers were used: miR-125b-1 (forward: 5′- CTGGT CACCTGATCCCATCT-;3′ and reverse: 5′-ATTGTTGCGCTCCTCTCAGT-3′; product size 217 bp), miR-125b-2 (forward: 5′-CCGCATCAAACCAGACT TTT-3′ and reverse: 5′-GGATGGGTCATGGTGAAAAC-3′; product size 228 bp), control 1 (forward: 5′-CAACGAATTTGGCTACAGCA-3′ and reverse: 5′- AGGGGTCTACATGGCAACTG-3′; product size 195 bp), and control 2 (forward: 5′- AGGAGCACGAGAGACTGAGG-3′ and reverse: 5′- CTGGCT GCTGTCATGTTCAG-3′; product size 232 bp). The data represent the means of three separate experiments. [file 1475-2867-13-21-S5.tiff]

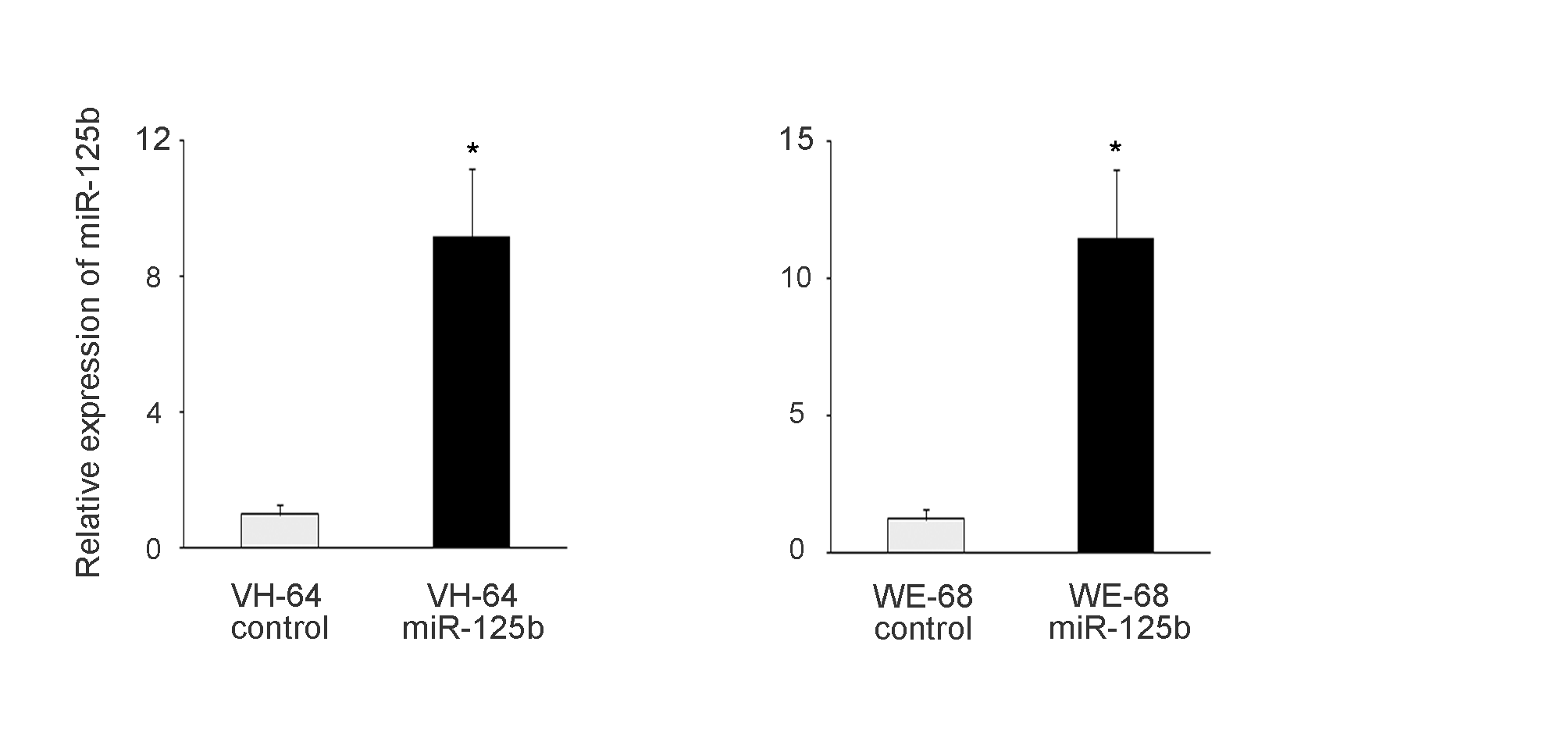

Supplement: Additional file 6: Figure S6 — The induction of miR-125b in parental EWS cells. The VH-64 or WE-68 parental cell lines were transfected with100 nM control or has-miR-125b miRNA Precursor. After 48 h incubation, the cells were collected, and qRT-PCR was performed to confirm the expression of miR-125b. [file 1475-2867-13-21-S6.tiff]

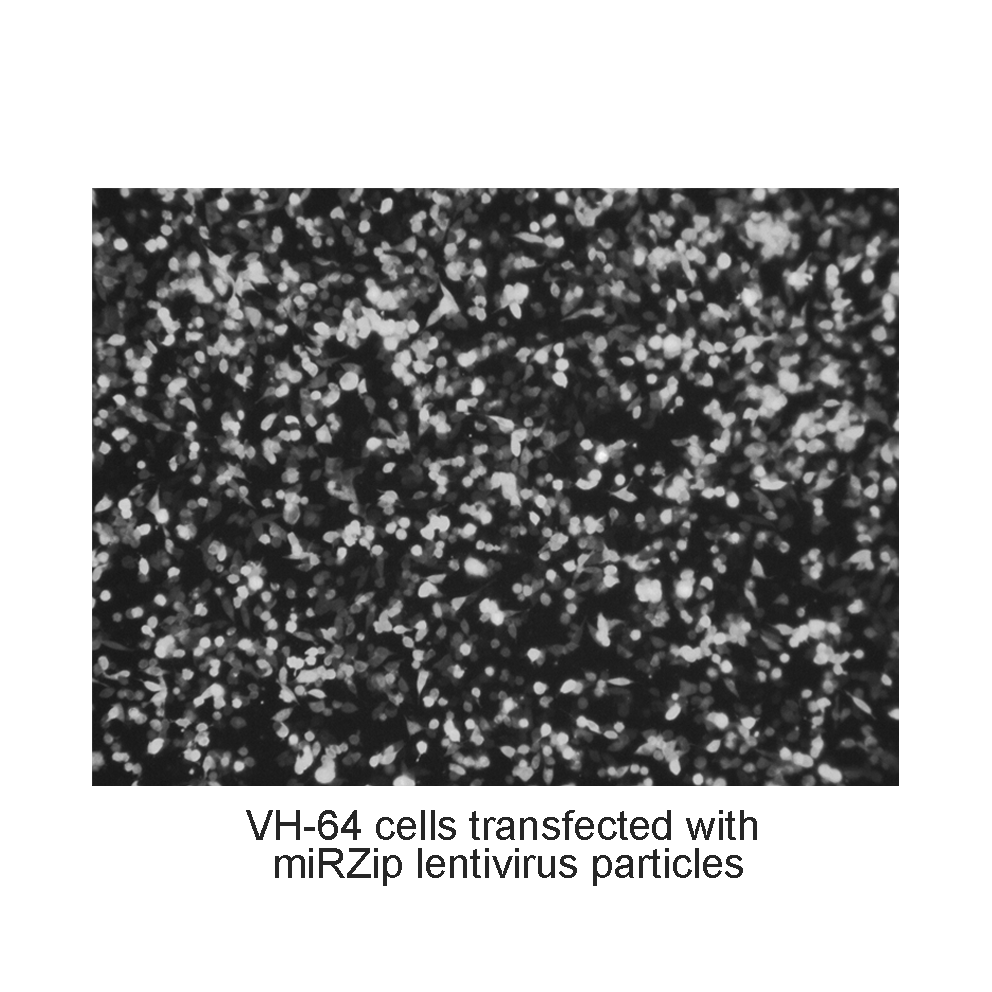

Supplement: Additional file 7: Figure S7 — The knockdown of miR-125b using the lentivirus. Cells were infected with the miRZip lentivirus construct, then the induction of the anti-miR-125b was confirmed by GFP-positivity in almost all of the cells. [file 1475-2867-13-21-S7.tiff]
